# Biomimicry Enhances Sequential Reactions of Tethered Glycolytic Enzymes, TPI and GAPDHS

**DOI:** 10.1371/journal.pone.0061434

**Published:** 2013-04-23

**Authors:** Chinatsu Mukai, Lizeng Gao, Magnus Bergkvist, Jacquelyn L. Nelson, Meleana M. Hinchman, Alexander J. Travis

**Affiliations:** 1 Baker Institute for Animal Health, College of Veterinary Medicine, Cornell University, Ithaca, New York, United States of America; 2 College of Nanoscale Science and Engineering, University at Albany, State University of New York, Albany, New York, United States of America; Brandeis University, United States of America

## Abstract

Maintaining activity of enzymes tethered to solid interfaces remains a major challenge in developing hybrid organic-inorganic devices. In nature, mammalian spermatozoa have overcome this design challenge by having glycolytic enzymes with specialized targeting domains that enable them to function while tethered to a cytoskeletal element. As a step toward designing a hybrid organic-inorganic ATP-generating system, we implemented a biomimetic site-specific immobilization strategy to tether two glycolytic enzymes representing different functional enzyme families: triose phosphoisomerase (TPI; an isomerase) and glyceraldehyde 3-phosphate dehydrogenase (GAPDHS; an oxidoreductase). We then evaluated the activities of these enzymes in comparison to when they were tethered via classical carboxyl-amine crosslinking. Both enzymes show similar surface binding regardless of immobilization method. Remarkably, specific activities for both enzymes were significantly higher when tethered using the biomimetic, site-specific immobilization approach. Using this biomimetic approach, we tethered both enzymes to a single surface and demonstrated their function in series in both forward and reverse directions. Again, the activities in series were significantly higher in both directions when the enzymes were coupled using this biomimetic approach versus carboxyl-amine binding. Our results suggest that biomimetic, site-specific immobilization can provide important functional advantages over chemically specific, but non-oriented attachment, an important strategic insight given the growing interest in recapitulating entire biological pathways on hybrid organic-inorganic devices.

## Introduction

A fundamental challenge in developing micro- and nanoscale hybrid material systems is determining how to interface biological components such as enzymes with inorganic surfaces without compromising enzymatic function. Surface attachment can affect multiple aspects of biocatalysis, and is therefore an important consideration in the design of hybrid organic-inorganic devices. For example, immobilization of enzymes can cause loss of function due to poor accessibility of substrate and/or limited ability to undergo needed conformational changes[1]–[3].

To address these difficulties in the context of working toward a bioenergy-producing platform technology, we are employing biomimicry, copying the design of the flagellum of mammalian sperm. These cells have evolved an elegant, high-throughput system for the local production of energy in the form of ATP in the flagellar principal piece. This region, which is the longest part of the flagellum, is devoid of mitochondria yet contains the majority of the dynein ATPases which consume the ATP [4], [5]. In this sub-cellular compartment, the sperm express the enzymes of glycolysis and array them along a solid cytoskeletal structure known as the fibrous sheath[6]–[9]. The ATP these enzymes produce locally powers both flagellar motility [10], as well as the signaling cascades believed to regulate different patterns of motility [11]. Several of the sperm’s glycolytic enzymes have been shown to differ from their somatic counterparts in that they have germ cell-specific domains that anchor them to the fibrous sheath[6], [12]–[15]. These splice variants allow sperm to have what is effectively a solid-state system of energy production, as opposed to having the enzymes exist primarily as soluble proteins as in most somatic cells. For the enzymes in which targeting domains have been identified, we hypothesize that the strategy of replacing or modifying these domains with a binding or affinity tag would allow tethering of these enzymes in a way that would maximize their function versus standard chemical approaches to binding.

Using our biomimetic strategy, we recently demonstrated that replacement of the germ cell-specific targeting domain of hexokinase (HK) with a hexahistidine tag (His) allowed the enzyme to be tethered to a surface through a defined binding site and with and retained function [16]. Using this approach, we tethered His-HK and the next glycolytic enzyme, glucose 6-phosphate isomerase (GPI), to a single inorganic support, and demonstrated their sequential enzymatic activities [16]. Moreover, we showed that the site-specific immobilization of GPI conferred a significant, 9-fold advantage in specific activity (and a 2-fold advantage in adsorption) versus GPI adsorbed with random orientation. To our knowledge, our demonstration of co-tethered HK and GPI was the first report of sequential enzymes from a biological pathway acting in series when co-tethered to a single support. Over the last decades, there have been many other demonstrations of systems with coupled enzymatic reactions, primarily utilizing enzyme combinations engineered to produce a desired product, such as coupling the activities of glucose oxidase and laccase to produce electricity from glucose [17]. In many cases, these enzymes are embedded to restrict their diffusion [18], [19]; in other cases, the enzymes are truly tethered, usually via chemical attachment strategies. Building upon these advancements, there is growing interest in rebuilding entire biological pathways on hybrid systems [20], [21].

Here, we evaluated whether a biomimetic attachment strategy would confer a comparative advantage in specific activity for another set of tethered enzymes that are part of the glycolytic pathway; namely, triose phosphoisomerase (TPI) and glyceraldehyde 3-phosphate dehydrogenase (GAPDHS). TPI is an isomerase that catalyzes the interconversion of glyceraldehyde 3-phosphate (GAP) and dihydroxyacetone phosphate (DHAP). GAPDHS is a male germ cell-specific isoform of the oxidoreductase, GAPDH, which phosphorylates glyceraldehyde 3-phosphate (using the cofactor NAD^+^) to produce 1,3-bisphosphoglycerate. These enzymes can catalyze both forward and reverse reactions; therefore, to demonstrate sequential, coupled activities, we ran the reactions in both directions. Importantly, we demonstrated that our biomimetic, site-specific immobilization conferred an advantage in specific activity versus a traditional conjugation approach that was chemically specific, but non-oriented. This is a critical point of comparison, as tethered enzyme function will dictate how glycolysis can best be recapitulated on hybrid organic-inorganic devices.

## Materials and Methods

### Generation of Recombinant TPI and GAPDHS

TPI and full length GAPDHS were cloned from mouse testes by RT-PCR (The primer sequences are available as Figure S1.). It has recently been found that male germ cells express one or more germ cell-specific isoforms of TPI, differing from the somatic isoform in the amino terminus (T. Ijiri and G. Gerton, *unpublished data*). Therefore, for TPI we engineered our binding tag on the amino terminus. The amino-terminal, germ cell-specific domain of GAPDHS is noteworthy both for its large size (105 amino acids) and its remarkable number of proline residues [22]. We refer to this domain as the “proline-rich domain” (PRD). During preliminary investigations, we made two variations of this protein, either modifying the PRD with a binding tag or replacing it with the binding tag. We found that the modified recombinant protein was expressed, but was highly insoluble and unable to be purified in a way that retained function. Replacement of the PRD was achieved by nested PCR using another forward primer. Constructs of cDNA for TPI and GAPDHS (minus the PRD) were cloned into the expression vector pcDNA4/His-Max TOPO TA (Invitrogen, Grand Island, NY), which added a hexahistidine repeat on the amino terminus of the expressed protein, followed by an enterokinase cleavage site and then the glycolytic enzyme’s sequence. Constructs were validated by sequencing and then were transfected into HEK293 FreeStyle cells (Invitrogen), using the FreeStyle Max™ transfection reagent (Invitrogen). The proteins were purified under native conditions from cell lysates 48 hr later using Ni-NTA agarose beads (Qiagen, Valencia, CA, USA). After the beads were washed, the recombinant proteins were eluted with 200 mM imidazole buffer and then were serially dialyzed with MOPS buffer (50 mM MOPS, 150 mM NaCl, pH 7.4) in order to avoid imidazole competitively binding to Ni-NTA groups during later surface attachment.

Protein concentrations were determined with the Micro BCA assay (Pierce, Rockford, IL), and purity of the samples was analyzed by SDS-PAGE, densitometric analysis of Coomassie staining, and immunoblotting. The primary antibodies used were as follows: mouse anti-His (1∶5000 dilution, Invitrogen), rabbit anti-GAPDHS (1∶1000 dilution, Protein Tech Group, IL), and rabbit anti-TPI (1∶2500 dilution, Abcam, Cambridge, MA). The secondary antibodies used were ECL anti-mouse IgG and ECL anti- rabbit IgG (Amersham, GE Healthcare, Piscataway, NJ), both conjugated with horseradish peroxidase.

### Assays of Individual Enzyme Function in Solution

Enzyme activities were measured by means of coupled enzyme reactions that led to either the production of NAD^+^ from NADH or the reduction of NAD^+^ to NADH. The rates of these reactions were measured as changes in absorbance at 340 nm using a spectrophotometer (Infinite 200 PRO and SAFIRE microplate reader, Tecan, Medford, MA). Unless otherwise noted, all biochemical reagents were obtained from Sigma (St. Louis, MO).

TPI activity was measured by the following coupled reactions, with the first catalyzed by His-TPI and the second with exogenous glycerophosphate dehydrogenase (GPDH):



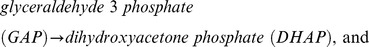





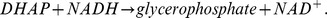



The reaction mixture contained 10 mM GAP, 0.4 mM NADH, and 1 U/ml GPDH in MOPS buffer. The Km for GAP was measured using a concentration range of 0.08–10 mM at a fixed NADH concentration (0.4 mM). One unit of TPI activity in this reaction was defined as the amount of enzyme that catalyzed the reduction of 1 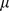
mol of GAP to DHAP per minute in a coupled system with GPDH.

GAPDHS activity was measured by the following coupled reactions, with the first catalyzed by exogenous phosphoglycerate kinase (PGK), and the second by His-GAPDHS:



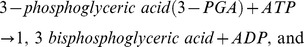





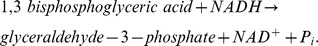



The reaction mixture contained 10 mM 3-PGA, 2 mM L-cysteine, 10 mM MgCl_2_, 0.4 mM NADH, 1 mM ATP and 3 U/ml PGK in MOPS buffer (50 mM MOPS, 150 mM NaCl, pH 7.4). The K_m_ for 3-PGA was measured using a concentration range of 0.08–10 mM at a fixed NADH concentration (0.4 mM). One unit of GAPDHS activity in this reaction was defined as the amount of enzyme that catalyzed the reduction of 1 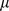
mol of 3-PGA to 1,3 bisphosphoglyceric acid per minute in a coupled system with PGK.

Measurements of activity were determined from slopes taken from within the linear range. The K_m_ and V_max_ for each enzyme were calculated using GraphPad Prism by non-linear regression analysis.

### Tethering of Enzymes to Inorganic Supports and Quantification of Activity While Bound

Site-specific immobilization was achieved by means of the His tag binding to a gold surface functionalized with nickel-nitrilotriacetic acid. Gold surfaces (10/100 nm Cr/Au deposited on silicon by electron-beam evaporation) were made functional with Ni-NTA for the immobilization of His-tagged proteins as described previously [23] with some modifications. Sulfuric acid (H_2_SO_4_), 30% hydrogen peroxide (H_2_O_2_), *N*-methyl-2-pyrrolidone, 16-mercaptohexadecanoic acid (MHA), ethanol, trifluoroacetic anhydride (TFA), triethylamine (TEA), *N*, *N*-dimethylformamide (DMF), tetrahydrofuran (THF), sodium hydroxide (NaOH), ethylenediaminetetraacetic acid (EDTA), nickel chloride (NiCl_2_), *N*, *N*-bis-(carboxymethyl)-L-lysine-hydrate (NTA), triethylene glycol, and all other chemical reagents were purchased from Sigma-Aldrich (St. Louis, MO) unless otherwise indicated.

Briefly, gold surfaces were cleaned by incubating in a mixture of 2∶1 H_2_SO_4_:H_2_O_2_ for 5 min, followed by rinsing with deionized water and then ethanol. Samples were immediately immersed in an ethanol solution containing 2 mM MHA and incubated overnight (∼16 hr). Control surfaces were incubated in ethanol without MHA. After incubation the surfaces were rinsed with ethanol, blown dry with high purity nitrogen (99.999%), and subsequently immersed in a solution of 100 mM TFA and 200 mM TEA in anhydrous DMF for 30 min. After the reaction (in which interchain anhydrides were formed), surfaces were rinsed thoroughly with DMF, blown dry with nitrogen, and immediately immersed in a solution containing 10 mM NTA, 10 mM triethylene glycol, and 7.5 mM NaOH at room temperature for 30 min. The surfaces were then rinsed with deionized water and blown dry under a stream of nitrogen. Ni-NTA activation was performed by immersing the gold surface in a solution of 40 mM NiCl_2_ at room temperature for 2 h. Finally, the functionalized gold surface was rinsed thoroughly with deionized water, dried with nitrogen, and cut into pieces (∼1 cm^2^) using a diamond-tipped stylus.

As a comparison for our strategy of site-specific immobilization, we tethered control enzymes on gold surfaces by means of classical carboxyl-amine covalent crosslinking. First, the gold surfaces were functionalized with carboxyl groups as described [16]. The gold surfaces were cleaned using a mixture of 3∶1 H_2_SO_4_: H_2_O_2_ for 5 min and rinsed with deionized water and ethanol. The surfaces were then immediately immersed in an ethanol solution containing 2 mM MHA and incubated overnight at room temperature. After incubation the surfaces were rinsed with ethanol and blown dry with N_2_. To activate carboxyl groups for covalent enzyme binding, the surfaces were subsequently immersed in a PBS solution (pH 7.2) containing 30 mM 1-ethyl-3- [3-dimethylaminopropyl] carbodiimide hydrochloride (EDC) and 10 mM N-hydroxysulfosuccinimide (NHS) (both from Thermo Fisher Scientific Inc., Rockford, IL) for 30 min. After the reaction, surfaces were rinsed thoroughly with PBS and MOPS (without drying).

Protein immobilization and determination of the amounts of immobilized proteins were performed as described previously [16]. Briefly, the proteins were incubated on a 1 cm^2^ gold surface for 15 min at ambient temperature for immobilization, and unbound proteins were washed off with MOPS buffer 3 times. Protein solutions and wash buffers were collected and protein concentrations were quantified with the Micro BCA assay. The amount of protein bound was calculated by subtraction.

We assayed the GAPDHS and TPI coupled reaction in series in both directions (from DHAP to 1,3 bis-PGA and from 3-PGA to glycerophosphate. For the forward reaction, the reaction mixture contained 10 mM DHAP, and 0.4 mM NAD^+^ in Glycine-phosphate buffer (50 mM glycine, 50 mM sodium monophosphate, pH 8.0). For the reverse reaction, the reaction mixture contained 10 mM 3-PGA, 2 mM L-cysteine, 10 mM MgCl_2_, 0.4 mM NADH, 1 mM ATP, 3U/ml PGK, 1 U/ml GPDH in MOPS buffer at pH 7.4.

## Results and Discussion

Whether used for in vivo or in vitro applications, biological effectors in hybrid organic-inorganic devices will predominantly rely upon ATP for energy. Glycolysis is a relatively simple, 10-step pathway in which ATP is produced from glucose. Although not as efficient as oxidative respiration in terms of ATP production per molecule of glucose, the cumulative size of all enzymes in the pathway is roughly equivalent to the molecular mass of just *one* respiratory complex. In addition, in terms of hybrid device engineering, glycolysis also has the comparative advantages of needing neither two membrane layers nor a proton-motive force in comparison to mitochondrial oxidative energy production [24]. Because of this comparative simplicity, we hypothesized that a biomimetic approach based on immobilized glycolytic enzymes from sperm would be advantageous in pursuing a long-term objective of designing an ATP-producing system. Here, we investigated the impact of different surface binding strategies on the tethered activities of TPI and GAPDHS.

**Figure 1:**
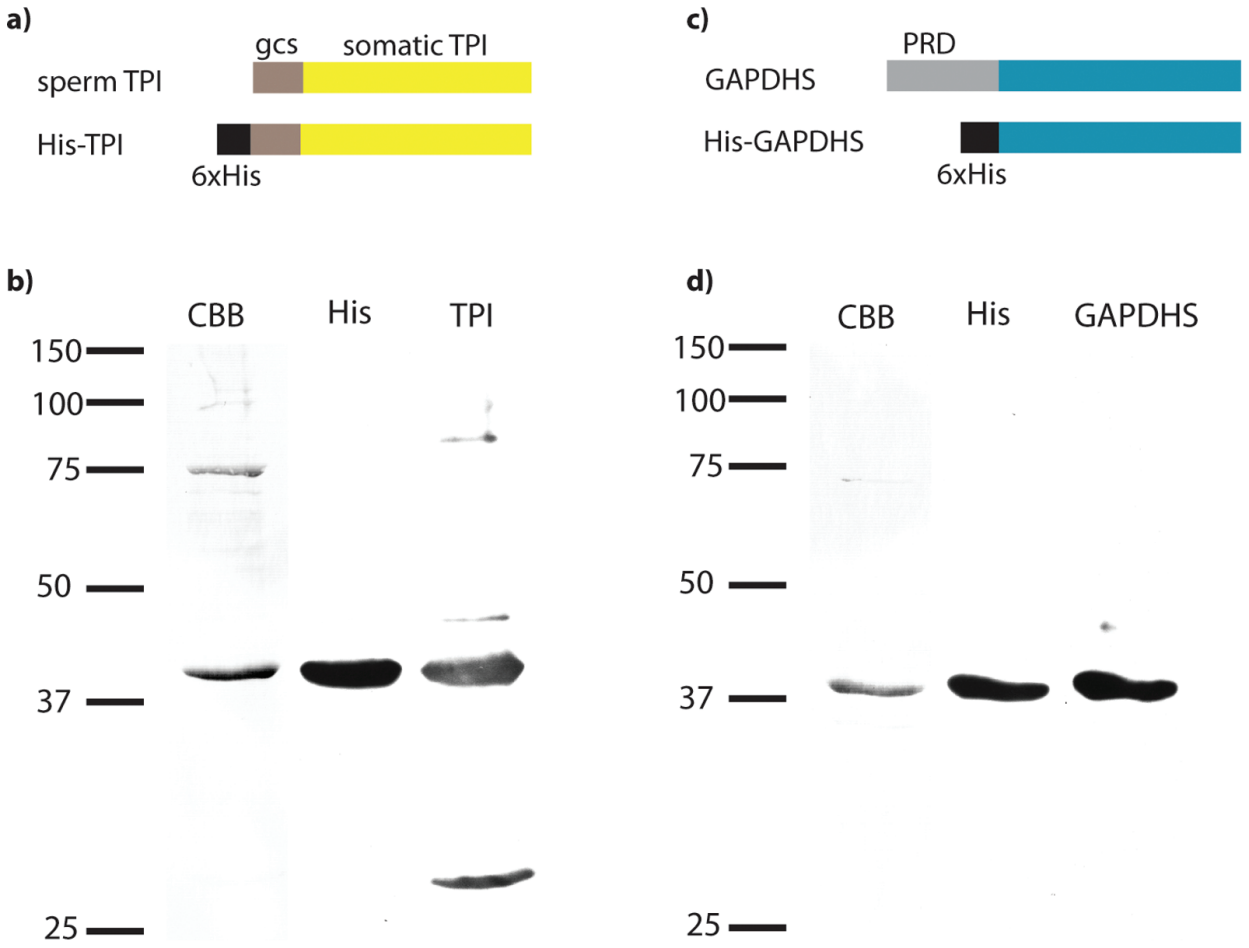
Design of recombinant proteins and verification of purified His-TPI and His-GAPDHS. a) A hexahistidine tag was introduced to modify the amino terminal, germ cell-specific domain (gcs) of sperm TPI. b) SDS-PAGE showing representative coomassie brilliant blue (CBB) protein staining and immunoblot analysis of purified His-TPI with antibodies against the His-tag (His) and the protein (TPI). c) A hexahistidine tag replaced the amino-terminal proline-rich domain (PRD) of GAPDHS. d) SDS-PAGE showing representative protein staining (CBB) and immunoblot analysis of purified His-GAPDHS with antibodies against the His-tag (His) and the protein (GAPDHS).

Both recombinant enzymes were repeatedly isolated at high purity (typically >80% for His-TPI, and >95% for His-GAPDHS), found to be immunoreactive with their appropriate antibodies, and to migrate at the expected molecular weights (Figure 1).

We first tested the activities of these purified enzymes in solution, determining optimal conditions of the reaction buffers. In solution, His-TPI had a V_max_ equal to 256 U/mg and a K_m_ equal to 0.092 mM (GAP) when tested in solution. His-GAPDHS demonstrated activity with a V_max_ equal to 5.2 U/mg and a K_m_ equal to 1.2 mM (3-PGA) (Table 1).

**Table 1:** Properties of recombinant proteins.

	V_ max_ (U/mg)	K_m_ (mM)
His-TPI	256±21	0.92±0.28
His-GAPDHS	5.2±0.68	1.2±0.55

Enzyme activities in solution were determined as described. K_m_ values are for the substrates, glyceraldehyde 3-phosphate (GAP) for TPI and 3-phosphoglyceric acid (3-PGA) for GAPDHS.

Several strategies can be used to immobilize enzymes onto surfaces; these include non-specific physical adsorption, covalent immobilization via functional groups on surface exposed amino acids, or various forms of bioaffinity such as avidin-biotin, antibody-ligand, or small peptide tags [25], [26]. For practical applications, proteins are frequently immobilized using methods that offer high surface coverage and stable anchoring; however, this approach often leads to random surface orientations that can have severe impacts on enzymatic function. In sperm, the enzymes of glycolysis are tethered to a cytoskeletal element via germ cell-specific domains. In creating our recombinant proteins, we replaced or modified these germ cell-specific sequences with His tags so that the enzymes would bind in a site-specific fashion. We hypothesized that the biomimetic use of the His tags to provide site-specific immobilization would result in improved specific activities in comparison with the carboxyl-amine chemistry, which although chemically specific, would result in tethered enzymes that had diverse orientations (although a subset would be bound by their terminal amine).

**Figure 2:**
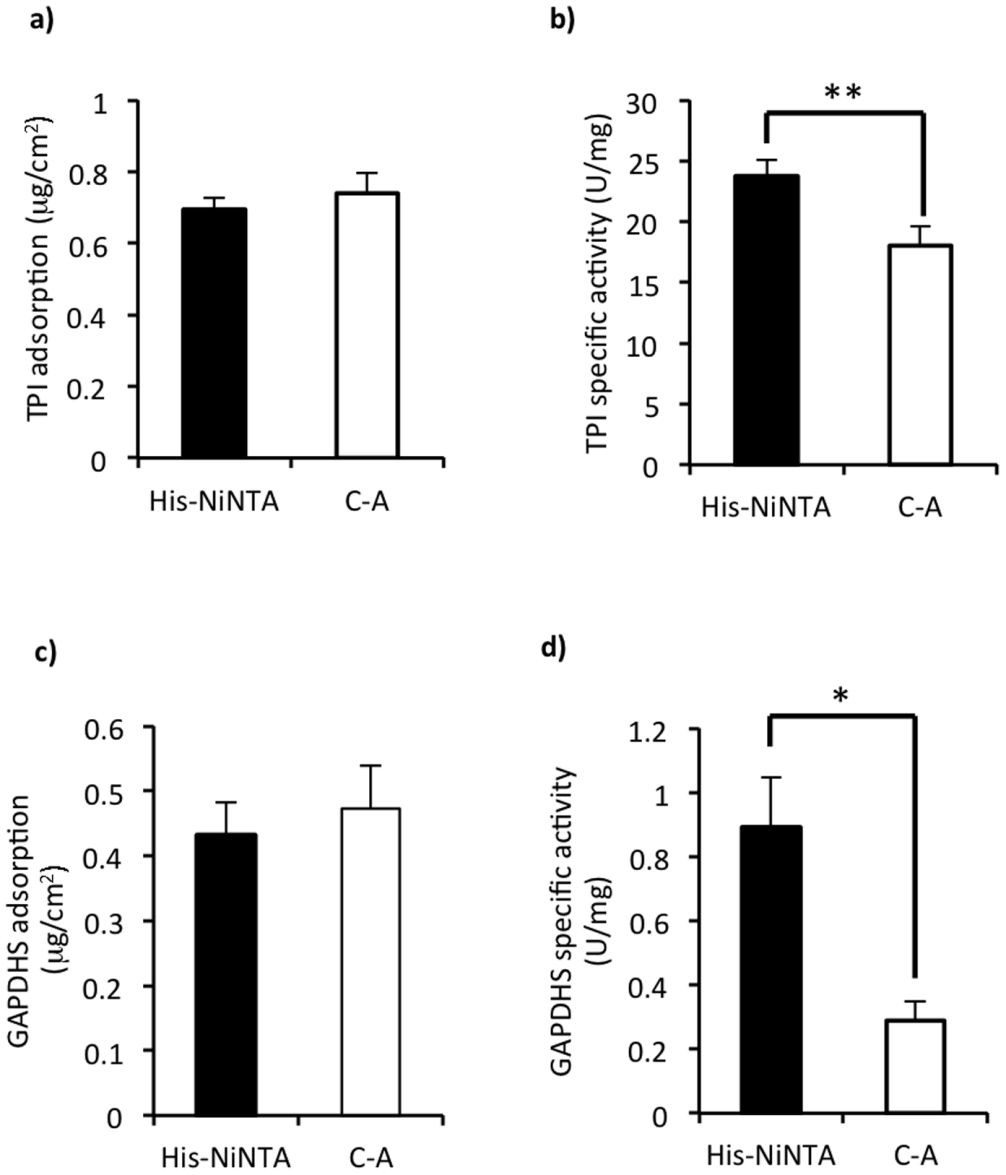
Site-specific immobilization improved specific activities of tethered enzymes. For both TPI and GAPDHS, site-specific immobilization using the His tag significantly improved enzyme specific activities versus carboxyl-amine binding. Although the total amounts of TPI (a) and GAPDHS (c) immobilized to carboxyl (C-A) or Ni-NTA activated surfaces were statistically identical, the specific activity of His-TPI bound to Ni-NTA was significantly higher than when bound via carboxyl-amine attachments (b; *p = 0.0143, n = 9). Similarly, the specific activity of His-GAPDHS was higher when bound to Ni-NTA versus carboxyl-amine binding (d; **p = 0.0234, n = 7).

We first modified gold surfaces with a carboxyl-terminated self-assembled monolayer. We then either functionalized that to have a nickel-nitrilotriacetic acid (Ni-NTA) activated surface for site-specific His-tag immobilization, or used carbodiimide chemistry to couple the enzymes via exposed amine groups. For both proteins, the two immobilization methods showed similar amounts of adsorbed protein (Figure 2a, and 2c). In both cases, the amount of recombinant proteins bound likely corresponded to monolayers (See Figure S4 for calculations regarding the number of molecules and surface coverage.).

Although carboxyl-amine binding provided the same amounts of protein adsorbed, the specific activities for both enzymes were significantly higher when they were immobilized via the His-tag (Figure 2b and 2d). We verified that the recombinant proteins with the His tags were indeed bound via that attachment by incubating the chips in a buffer containing imidazole (400 mM). Over 90% of the tethered proteins’ activities were lost in response due to the removal of protein (n = 9, p = 0.0002). In comparison, when the same wash was performed on the carboxyl-amine-bound enzymes, they lost approximately 30% of their activity, which was not a significant difference (n = 9, p = 0.1962) (data not shown). These data verified that the biomimetic, site-specific immobilization conferred by the binding tag provided a significant improvement in terms of enzyme specific activity. In conjunction with previous data [16], we have now shown a comparative advantage for biomimetic attachment for three broad functional classes of enzymes represented by hexokinase (a transferase), GPI and TPI (isomerases), and GAPDHS (an oxidoreductase).

To investigate the enzymes’ ability to function in series, we combined the recombinant His-TPI and His-GAPDHS in solution and found that they performed sequential reactions in both directions. This demonstrated that they did not interfere with each other’s functions (data not shown). We examined the optimal conditions for GAPDHS forward direction activity in solution (Figure S2), and found that both commercial GAPDH and recombinant GAPDHS showed higher activity in basic glycine-phosphate buffer (pH 8.5). In solution, the coupled TPI and GAPDHS reaction also showed higher activity with basic pH, with little difference between pH 8.0–8.5 (Figure S3). Because future plans involve integration of all the glycolytic enzymes having different pH optima, we utilized glycine-phosphate buffer with pH 8.0 to assay the tethered sequential reactions.

**Figure 3:**
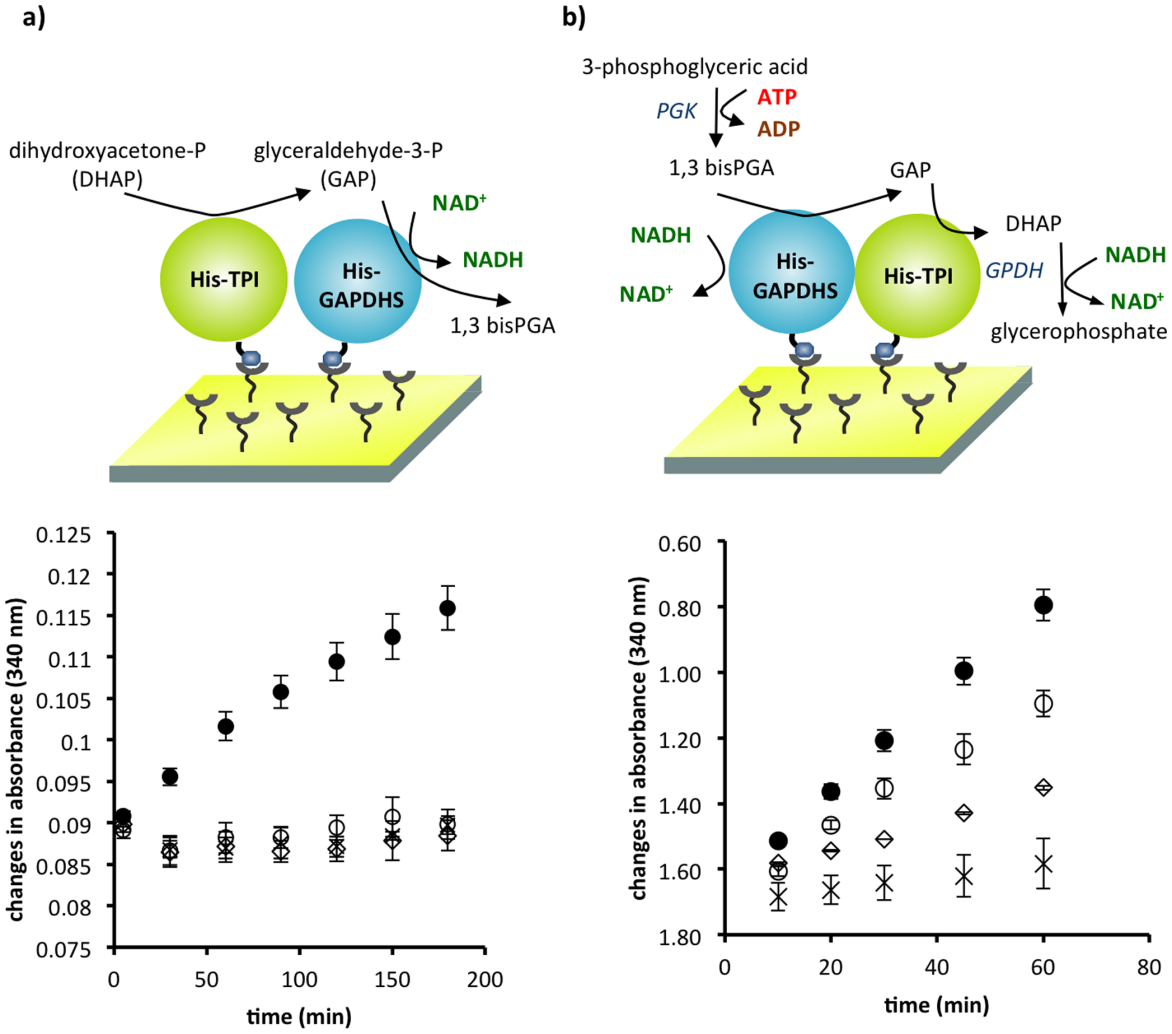
Activity of His-TPI and His-GAPDHS in series on a single inorganic support. a) Schematic diagram of the forward TPI-GAPDHS coupled reaction (top), with corresponding assay results (bottom). b) Schematic diagram of the reverse GAPDHS-TPI coupled reaction (top), with corresponding assay results (bottom). Symbols represent both TPI and GAPDHS on the chip (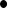), TPI alone on the chip (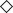), GAPDHS alone on the chip (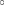) and control chips having no attached protein, but the complete reaction mixtures (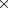). Activity for the forward reaction differed significantly from the blank (p = 0.0019), and coupled activities for controls with each enzyme by itself did not differ significantly from the blank. Comparisons of activity for the reverse reaction showed significant differences for the coupled reaction versus the blank (p = 0.0139), versus GAPDHS alone (p = 0.0123), and versus TPI alone (p = 0.0241). Activity for GAPDHS alone versus TPI alone was not significantly different. Statistics were performed with paired t tests. n = 9 for chips with both enzymes, n = 3 for single enzyme and n = 5 for blank controls. Mean values are plotted with SE.

To perform the sequential reaction and associated controls, 3–5 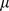
g of either His-TPI, His-GAPDHS, or both proteins (total) were applied to individual chips (1 cm^2^, Ni-NTA surface). For chips getting both proteins, a solution containing both His-TPI and His-GAPDHS was prepared with the enzymes in a 1∶1 molar ratio. Given the disparity in specific activities between the enzymes, we also prepared chips with a 25 GAPDHS : 1 TPI ratio and surprisingly did not see any improvement in efficiency of the coupled tethered reaction (data not shown; we are now performing experiments to determine what factors regulate the efficiency of the coupled reaction, which are beyond the scope of the current experimental comparisons.). We assessed the ability of the tethered enzymes to carry out sequential enzymatic reactions in both forward and reverse directions. In the forward direction, co-tethered His-TPI and His-GAPDHS showed sequential activities whereas surfaces with only a single species of protein did not show any activity (Figure 3a). This finding confirmed that our biochemical detection method was indeed specific for the sequential reactions. In the reverse direction, GAPDHS can convert NADH to NAD^+^ without TPI activity. As expected in this direction, the chip with GAPDHS alone showed a change in absorbance, though lower than when both enzymes were present and two reactions each produced NAD^+^ (Figure 3b). Chips with TPI alone showed a minor change in absorbance; however, the change was significantly lower than surfaces with both enzymes or GAPDHS alone.

**Figure 4:**
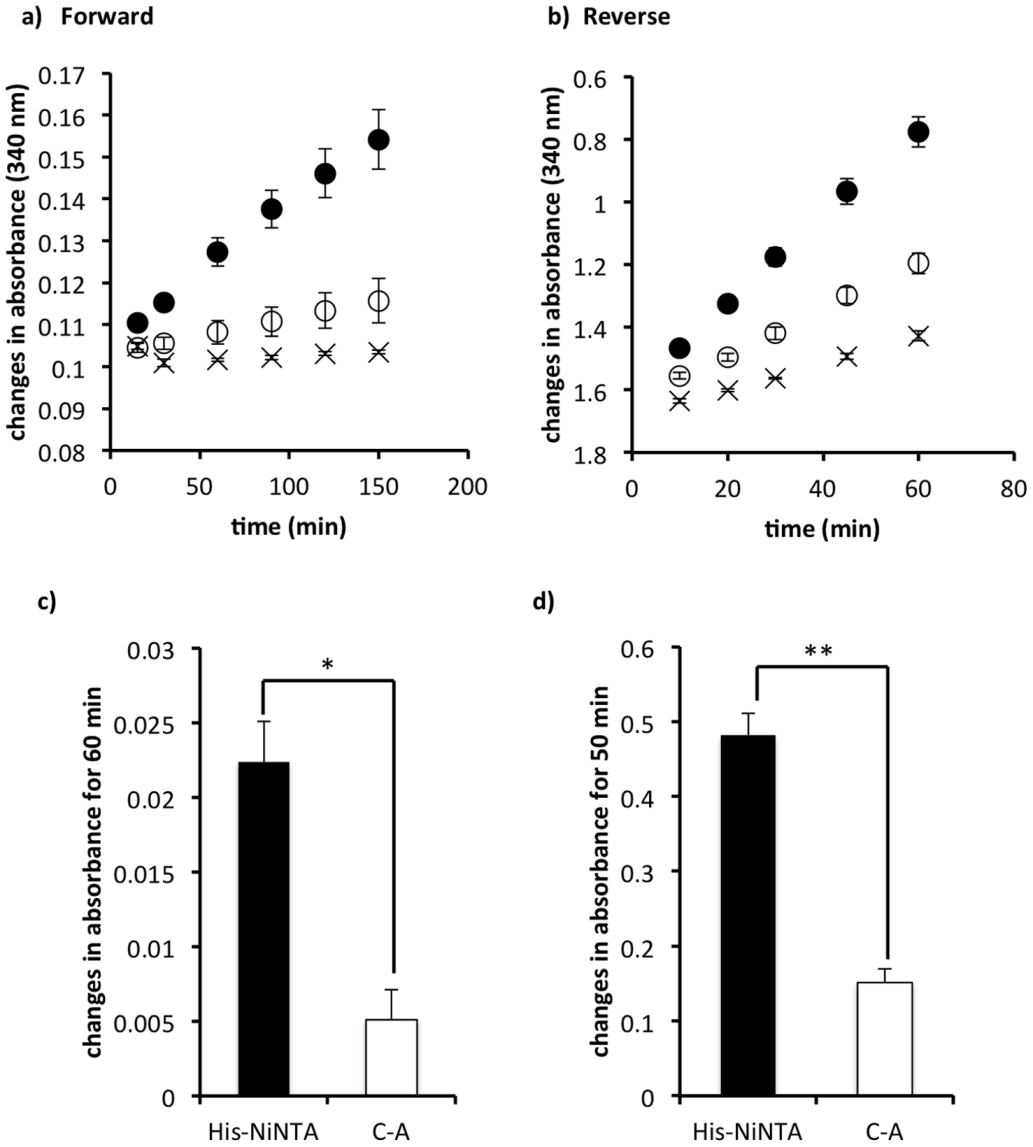
Site-specific immobilization improved the coupled reaction of tethered enzymes. a) Forward TPI-GAPDHS coupled reaction. b) Reverse GAPDHS-TPI coupled reaction. Both enzymes were tethered via their His tags and site-specific immobilization (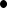); both enzymes were tethered via carboxyl-amine binding (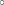); control chips had no attached protein, but the complete reaction mixtures (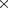) [n = 9 (a), n = 12 (b); mean values are plotted with SE]. c) Comparison of forward TPI-GAPDHS activities at the 50 min timepoint calculated from (a). d) Comparison of reverse GAPDHS-TPI activities at the 60 min timepoint calculated from (b). Site-specific immobilization of His-NiNTA showed significantly higher activity (*p = 0.0001 ** p<0.001).

We also compared the impact of immobilization strategy on the activity of the sequential reaction (Figure 4). The coupled activity of enzymes bound via site-specific immobilization was significantly higher compared to that of proteins tethered via carboxyl-amine binding. In the forward direction, coupled activity was 3.2-fold higher using the biomimetic strategy (Figure 4c), and 4.4-fold higher in the reverse direction (Figure 4d). These data demonstrated that the benefits of site-specific immobilization extended beyond the activity of single proteins to multi-enzyme pathways as well.

Our data demonstrated that a biomimetic approach for site-specific immobilization of enzymes could have significant value in the design of hybrid devices. We did not observe any difference in the amounts of proteins adsorbed in comparison with classical chemical binding (Figure 2a and 2c), and we observed significant improvements in enzyme specific activity (Figure 2b. 2d, 4c, and 4d). Importantly, even when tethered to a large, planar surface that would be predicted to impart challenges regarding the diffusion and approach of metabolic intermediates, the enzymes worked. Ultimately, to generate energy in the form of ATP on implantable medical devices, TPI and GAPDHS would exist as part of the complete, multi-step glycolytic pathway. The current data double the progress toward that goal, with 4 enzymes (HK, GPI, TPI, and GAPDHS) showing tethered activity in two paired reactions [16]. A complete glycolytic assemblage would benefit from the fact that glucose is freely available in the circulation. Easy access to its metabolic substrate is a significant advantage to systems integrating glycolysis in comparison to several alternative energy-generating systems that have been described, which rely either on exogenous supplies of photons [27]or the metabolic intermediate, phosphoenolpyruvate [28].

Although our data have been specific to tethered glycolytic enzymes, they suggest more broadly that efforts to tether other functional enzymes might benefit from a biological strategy versus a chemical approach. For example, highly polarized cells such as sperm, neurons, or retinal cells might provide additional insights into device engineering through their precise compartmentalization of cellular functions.

### Conclusion

Our data demonstrated the activity in series of sequential glycolytic enzymes, TPI and GAPDHS, tethered to a solid support. Comparison of the specific activities of the enzymes tethered via site-specific immobilization versus specific, but non-oriented chemical immobilization confirmed the advantage of using a biomimetic strategy based on the arrangement of glycolytic enzymes in the sperm flagellum. These data provide an important example of how biomimicry can improve the functional efficiency of hybrid organic-inorganic devices. Moreover, they build upon our previous demonstration of co-tethered HK and GPI activities, doubling progress toward recapitulating glycolysis on hybrid organic-inorganic devices, an important step in the design of an energy-producing platform technology capable of generating ATP from glucose.

## Supporting Information

Figure S1 **Primer sequences for TPI and GAPDHS.**
(DOC)

Figure S2 **Optimization of the assay buffers for GAPDH and GAPDHS forward reactions.**
(DOC)

Figure S3 **Effect of pH on the activity of the coupled TPI-GAPDHS forward reaction.**
(DOC)

Figure S4 **Estimated calculations for surface coverage of tethered enzymes on chips.**
(DOC)

## References

[1] Fischer T, Hess H (2007) Materials chemistry challenges in the design of hybrid bionanodevices: supporting protein function within artificial environments. Journal of Materials Chemistry 17: 943–951.

[2] Halliwell CM, Morgan G, Ou CP, Cass AE (2001) Introduction of a (poly)histidine tag in L-lactate dehydrogenase produces a mixture of active and inactive molecules. Anal Biochem 295: 257–261. 11488630 10.1006/abio.2001.5182

[3] Tachibana S, Suzuki M, Asano Y (2006) Application of an enzyme chip to the microquantification of l-phenylalanine. Anal Biochem 359: 72–78. 17046706 10.1016/j.ab.2006.09.006

[4] Inaba K (2011) Sperm flagella: comparative and phylogenetic perspectives of protein components. Mol Hum Reprod 17: 524–538. 21586547 10.1093/molehr/gar034

[5] Fawcett DW (1970) A comparative view of sperm ultrastructure. Biol Reprod 2 Suppl 290–127. 12254595

[6] Cao W, Gerton GL, Moss SB (2006) Proteomic profiling of accessory structures from the mouse sperm flagellum. Mol Cell Proteomics 5: 801–810. 16452089 10.1074/mcp.M500322-MCP200

[7] Krisfalusi M, Miki K, Magyar PL, O’Brien DA (2006) Multiple glycolytic enzymes are tightly bound to the fibrous sheath of mouse spermatozoa. Biol Reprod 75: 270–278. 16687649 10.1095/biolreprod.105.049684

[8] Storey BT, Kayne FJ (1975) Energy metabolism of spermatozoa. V. The Embden-Myerhof pathway of glycolysis: activities of pathway enzymes in hypotonically treated rabbit epididymal spermatozoa. Fertil Steril 26: 1257–1265. 803042

[9] Westhoff D, Kamp G (1997) Glyceraldehyde 3-phosphate dehydrogenase is bound to the fibrous sheath of mammalian spermatozoa. J Cell Sci 110 (Pt 15): 1821–1829. 10.1242/jcs.110.15.1821 9264469

[10] Mukai C, Okuno M (2004) Glycolysis plays a major role for adenosine triphosphate supplementation in mouse sperm flagellar movement. Biol Reprod 71: 540–547. 15084484 10.1095/biolreprod.103.026054

[11] Travis AJ, Jorgez CJ, Merdiushev T, Jones BH, Dess DM, et al (2001) Functional relationships between capacitation-dependent cell signaling and compartmentalized metabolic pathways in murine spermatozoa. J Biol Chem 276: 7630–7636. 11115497 10.1074/jbc.M006217200

[12] Travis AJ, Sui D, Riedel KD, Hofmann NR, Moss SB, et al (1999) A novel NH(2)-terminal, nonhydrophobic motif targets a male germ cell-specific hexokinase to the endoplasmic reticulum and plasma membrane. J Biol Chem 274: 34467–34475. 10567428 10.1074/jbc.274.48.34467

[13] Mori C, Nakamura N, Welch JE, Gotoh H, Goulding EH, et al (1998) Mouse spermatogenic cell-specific type 1 hexokinase (mHk1-s) transcripts are expressed by alternative splicing from the mHk1 gene and the HK1-S protein is localized mainly in the sperm tail. Mol Reprod Dev 49: 374–385. 9508088 10.1002/(SICI)1098-2795(199804)49:4<374::AID-MRD4>3.0.CO;2-K

[14] Travis AJ, Foster JA, Rosenbaum NA, Visconti PE, Gerton GL, et al (1998) Targeting of a germ cell-specific type 1 hexokinase lacking a porin-binding domain to the mitochondria as well as to the head and fibrous sheath of murine spermatozoa. Mol Biol Cell 9: 263–276. 9450953 10.1091/mbc.9.2.263 PMC25249

[15] Welch JE, Schatte EC, O’Brien DA, Eddy EM (1992) Expression of a glyceraldehyde 3-phosphate dehydrogenase gene specific to mouse spermatogenic cells. Biol Reprod 46: 869–878. 1375514 10.1095/biolreprod46.5.869

[16] Mukai C, Bergkvist M, Nelson JL, Travis AJ (2009) Sequential reactions of surface- tethered glycolytic enzymes. Chem Biol 16: 1013–1020. 19778729 10.1016/j.chembiol.2009.08.009 PMC4051345

[17] Zebda A, Gondran C, Le Goff A, Holzinger M, Cinquin P, et al (2011) Mediatorless high-power glucose biofuel cells based on compressed carbon nanotube-enzyme electrodes. Nat Commun 2: 370. 21712818 10.1038/ncomms1365 PMC3156815

[18] Onda M, Lvov Y, Ariga K, Kunitake T (1996) Sequential actions of glucose oxidase and peroxidase in molecular films assembled by layer-by-layer alternate adsorption. Biotechnol Bioeng 51: 163–167. 18624325 10.1002/(SICI)1097-0290(19960720)51:2<163::AID-BIT5>3.0.CO;2-H

[19] Pescador P, Katakis I, Toca-Herrera JL, Donath E (2008) Efficiency of a bienzyme sequential reaction system immobilized on polyelectrolyte multilayer-coated colloids. Langmuir 24: 14108–14114. 19360959 10.1021/la8027435

[20] Keighron JD, Keating CD (2011) Towards a minimal cytoplasm. In: Luisi PL, P.Stano, editors. The minimal cell: the Biophysics of Cell Compartment and the Origin of Cell Functionality: Springer Science. pp.3–32.

[21] Keighron JD, Keating CD (2010) Enzyme:nanoparticle bioconjugates with two sequential enzymes: stoichiometry and activity of malate dehydrogenase and citrate synthase on Au nanoparticles. Langmuir 26: 18992–19000. 21114258 10.1021/la1040882 PMC3012446

[22] Bunch DO, Welch JE, Magyar PL, Eddy EM, O’Brien DA (1998) Glyceraldehyde 3-phosphate dehydrogenase-S protein distribution during mouse spermatogenesis. Biol Reprod 58: 834–841. 9510974 10.1095/biolreprod58.3.834

[23] Yan L, Marzolin C, Terfort A, Whitesides GM (1997) Formation and reaction of interchain carboxylic anhydride groups on self-assembled monolayers on gold. Langmuir 13: 6704–6712.

[24] Voet D, Voet JG (2010) Electron Transport and oxidative phosphorylation. Biochemistry. 4 ed: Wiley. pp.823–862.

[25] Rusmini F, Zhong Z, Feijen J (2007) Protein immobilization strategies for protein biochips. Biomacromolecules 8: 1775–1789. 17444679 10.1021/bm061197b

[26] Jonkheijm P, Weinrich D, Schroder H, Niemeyer CM, Waldmann H (2008) Chemical strategies for generating protein biochips. Angew Chem Int Ed Engl 47: 9618–9647. 19025742 10.1002/anie.200801711

[27] Luo TJ, Soong R, Lan E, Dunn B, Montemagno C (2005) Photo-induced proton gradients and ATP biosynthesis produced by vesicles encapsulated in a silica matrix. Nat Mater 4: 220–224. 15696172 10.1038/nmat1322

[28] Du YZ, Hiratsuka Y, Taira S, Eguchi M, Uyeda TQ, et al.. (2005) Motor protein nano-biomachine powered by self-supplying ATP. Chem Commun (Camb): 2080–2082. 10.1039/b500327j 15846406

