# Supplementary material for: Biomimicry Enhances Sequential Reactions of Tethered Glycolytic Enzymes, TPI and GAPDHS
Source: PLoS One. 2013 Apr 23;8(4):e61434. doi: 10.1371/journal.pone.0061434 (PMC3634084; doi:10.1371/journal.pone.0061434)
Supplement: Figure S1 — Primer sequences for TPI and GAPDHS. (DOC) [file pone.0061434.s001.doc]

**Biomimicry enhances sequential reactions of tethered glycolytic enzymes, TPI and GAPDHS**

Chinatsu Mukai, Lizeng Gao, Magnus Bergkvist, Jacquelyn L. Nelson, Meleana M. Hinchman and Alexander J. Travis

**Supporting Information**.

**Figure S1. Primer sequences for TPI and GAPDHS**

TPI forward: 5’- GCCAGAACGTCGAAACACC

TPI reverse: 5’- CGTGGATTAGGCCACAAGAT

GAPDHS full length forward: 5’- ATGTCGAGACGTGACGTGGTCCTTACC

GAPDHS nested forward: 5’- CTGACAGTGGGTATCAATGGATTTGG

GAPDHS reverse: 5’- TTACTTCTCTCGGCTAAACATGTAGCG
