# Supplementary material for: Biomimicry Enhances Sequential Reactions of Tethered Glycolytic Enzymes, TPI and GAPDHS
Source: PLoS One. 2013 Apr 23;8(4):e61434. doi: 10.1371/journal.pone.0061434 (PMC3634084; doi:10.1371/journal.pone.0061434)
Supplement: Figure S2 — Optimization of the assay buffers for GAPDH and GAPDHS forward reactions. (DOC) [file pone.0061434.s002.doc]

**Figure S2. Optimization of the assay buffers for GAPDH and GAPDHS forward reactions**

To optimize the forward reactions for GAPDH and GAPDHS, we performed activity assays with different media and different pH conditions. Initial efforts to use MOPS-NaCl buffer revealed that it did not support activity for either commercial GAPDH or recombinant GAPDHS (panel A for GAPDH, panel C for GAPDHS). In contrast, use of a glycine-based phosphate buffer (50 mM glycine, 50 mM sodium monophosphate) supported much higher activity for both commercial GAPDH (panel B) and recombinant GAPDHS (panel D). Varying pH between 7-8.5 revealed that activity increased with the more basic pH conditions for both proteins. Bars represent standard error from triplicates. 0.5 U of Sigma GAPDH (calculated from product certificate) was used for each assay as commercial enzyme, and 0.5 $\mu$g of His-GAPDHS was used for each assay.
