# Supplementary material for: Biomimicry Enhances Sequential Reactions of Tethered Glycolytic Enzymes, TPI and GAPDHS
Source: PLoS One. 2013 Apr 23;8(4):e61434. doi: 10.1371/journal.pone.0061434 (PMC3634084; doi:10.1371/journal.pone.0061434)
Supplement: Figure S3 — Effect of pH on the activity of the coupled TPI-GAPDHS forward reaction. (DOC) [file pone.0061434.s003.doc]

**Figure S3. Effect of pH on the activity of the coupled TPI-GAPDHS forward reaction**

Based on the impacts of the medium and pH on GAPDHS activity, we tested the glycine-phosphate buffer for its potential impacts on the TPI-GAPDHS coupled reaction in solution. Coupled activities were highly similar for pH 8.0-8.5, and higher than when tested at pH 7 or 7.5.
