# Supplementary material for: Biomimicry Enhances Sequential Reactions of Tethered Glycolytic Enzymes, TPI and GAPDHS
Source: PLoS One. 2013 Apr 23;8(4):e61434. doi: 10.1371/journal.pone.0061434 (PMC3634084; doi:10.1371/journal.pone.0061434)
Supplement: Figure S4 — Estimated calculations for surface coverage of tethered enzymes on chips. (DOC) [file pone.0061434.s004.doc]

Figure S4. **Estimated calculations for surface coverage of tethered enzymes on chips**

When applied individually, His-TPI (MW 37512) and His-GAPDHS (MW 39959) typically had protein adsorptions of 0.65 $\mu$g/cm^2^ and 0.45 $\mu$g/cm^2^ protein adsorption, respectively. 0.65 $\mu$g of His TPI is equivalent to 0.017 nmole, which is itself equal to 1.02 x10^13^ molecules calculated by using Avogadro’s constant. Similarly, 0.45 $\mu$g of His-GAPDHS is equivalent to 0.011 nmole, which is equal to 0.66 x10^13^ molecules.

Based on their molecular weights, both molecules can be estimated as globular molecules having diameters of approximately 3 nm[^1^](#_ENREF_1). A 1 cm^2^ surface can hold approximately 1.11 x10^13^ molecules of 3 nm diameter. Thus, our tethered enzymes were at a density consistent with formation of a monolayer on the chips.

1. Erickson, H. P., Size and shape of protein molecules at the nanometer level determined by sedimentation, gel filtration, and electron microscopy. *Biol Proced Online* **2009,** 11, 32-51.
